# Cryopreservation of individually selected sperm: methodology and case report of a clinical pregnancy

**DOI:** 10.1007/s10815-012-9733-y

**Published:** 2012-03-03

**Authors:** Nina Desai, Jeffrey Goldberg, Cynthia Austin, Edmund Sabanegh, Tommaso Falcone

**Affiliations:** 1 Department of OB-GYN, Cleveland Clinic Fertility Center, Beachwood, OH USA; 2 Glickman Urological Institute, Cleveland Clinic, Cleveland, OH USA

**Keywords:** Epidydimal, Testicular, Sperm, Cryopreservation, Single sperm freezing, Hamster zona, HSV straw, Closed carrier, ICSI

## Abstract

**Objective:**

To describe a new technique for freezing individually isolated spermatazoa from testicular biopsies, epididymal aspirates and oligospermic semen samples

**Methods:**

Samples were evaluated for the presence of motile sperm before cryopreservation. Motile or twitching sperm were isolated with an ICSI needle for single sperm cryopreservation. Selected sperm were loaded on the High Security Straw (HSV; Irvine Scientific; Irvine,CA), in ~0.5 µl of fluid to facilitate recovery. The sample was also frozen using conventional methodology in cryovials (100–1000 µl aliquots). In both freezing techniques, the samples were slow cooled. Test-yolk buffer-glycerol (Irvine) was used as the cryoprotectant. Test-thaws were performed to assess sperm recovery and motility.

**Results:**

Six men with azoospermia had single sperm cryopreservation, as well as freezing aliquots of their testicular or epididymal sperm in traditional cryovials. In addition, two men with oligospermia also had individual sperm selected and frozen. In all 8 cases, the ~0.5 µl of fluid containing sperm was quite easily unloaded from the HSV straw during thawing. The percent sperm recovery ranged from 33% to 100%. Motility was evident in all but one sample. In six cases, the sperm were used for intracytoplasmic sperm injection of mature oocytes. Fertilization occurred in all but one case. In this study, we report the first clinical pregnancy with this technique. This pregnancy was remarkable in that a single motile sperm identified and selected in the initial testicular preparation was successfully frozen. We were able to subsequently recover this sperm, fertilize an oocyte and the resultant embryo gave rise to a live birth. The methodology described in this preliminary report offers a new modality for sequestering small numbers of sperm. It may be particularly useful in cases involving severe impairment of spermatogenesis, where extensive screening may be necessary to find a few viable sperm.

## Introduction

In vitro fertilization combined with intracytoplasmic sperm injection (ICSI) have provided new treatment options for azoospermic men through surgical or percutaneous isolation of sperm from the testis or epididymis. Oocyte injection with sperm isolated from testicular biopsies or epididymal aspirates have been quite effective in establishing pregnancies [9]. The first live births with epididymal and testicular sperm were reported by Silber et al [11, 12], respectively. The severity of spermatogenesis impairments that clinicians can treat has increased through advances in both surgical extraction methodology as well as laboratory techniques.

To avoid the expense and complications resulting from repeated surgical sperm retrievals, cryopreservation of sperm is necessary. This has proven to be extremely challenging in cases of non-obstructive azoospermia due to very limited numbers of viable sperm in epidiymal and testicular samples. Conventional cryopreservation techniques are inadequate for preserving individually selected sperm. The size of the vessel, freezing volume and adherence of sperm to vessel walls make them especially unsuitable for freezing tiny aliquots containing 10–30 sperm. The extremely innovative methods described for cryopreservation of small quantities of sperm in evacuated hamster zonae [3], as well as human zonae [7], clearly demonstrate that this is logistically possible. Moreover, despite the severity of the spermatogenesis impairment in azoospermia patients, the thawed sperm were in fact capable of producing healthy infants. Yet it is equally clear that achieving the same result using less cumbersome methodology and not involving the use of evacuated animal or human oocytes as a vessel for storing and cryopreserving the sperm would be extremely advantageous. Alternate methods for single sperm freezing have been attempted over the years but have had their limitations [1]. Sperm storage in microdrops placed on culture dishes [2, 10] or in ICSI pipettes has been tried but long term storage in these vessels is not practical. Others have tried encapsulation in alginate beads [6] as well as injection in to algae spheres [8] but neither of these methods were ideal and/or convenient for handling a few individual sperm.

We therefore began exploring different methodologies for storing small numbers of individually selected sperm. We previously described the use of the cryoloop to freeze individually isolated sperm in miniscule volumes of fluid measuring less than one microliter [5]. More recently, we have developed a unique method that allows us to use the HSV (High Security Straw) for storage of individually selected sperm. This new device is FDA cleared for embryo cryopreservation and is easily adaptable for storing miniscule volumes of sperm. In this sealed system, there is no direct contact of the sperm with liquid nitrogen. This novel freezing technique, and our first clinical pregnancy, are described in this report.

## Materials and methods

### Patient selection/study design

Single sperm cryopreservation was undertaken for six patients with azoospermia, following testicular biopsy or epididymal sperm aspiration. In two additional instances, the procedure was utilized for oligospermic semen specimens. IRB approval was obtained for data extraction and all techniques were part of routine laboratory procedure. The methodology for preparation and evaluation of testicular tissue and epididymal samples has been previously described in detail [4]. Samples were assessed for the presence of sperm using an inverted microscope at 300× magnification.

The Single Sperm Freezing (SSF) technique was applied when low numbers of motile sperm were observed in an aliquot of the initial fresh specimen. In such cases, an ICSI embryologist attempted to collect 10–20 of these sperm for freezing on HSV straws (Irvine Scientific; Irvine,CA). If sperm were sufficient, a second straw was prepared for a test-thaw. The remaining specimen was then frozen in conventional vials.

### Sperm cryopreservation-conventional method

Aliquots of epididymal aspirate, testicular homogenate or semen sample were diluted 1:1 with test yolk buffer-glycerol cryoprotectant (TYB-GLY; Irvine Scientific). Based on the number of sperm viewed, 1–4 vials, along with a small test sample were frozen per patient. Vials were vapor frozen for 30–60 min prior to immersion in liquid nitrogen.

Frozen samples were thawed at room temperature, the morning of the patient’s egg retrieval. Cryoprotectant was removed by centrifugation for 10 min at 300 g. The sperm pellet was re-suspended in 50–100 μl of modified HTF (mHTF; Irvine Scientific) with 10 mg/ml of HSA (Sage; Pasadena, CA).

### Single sperm cryopreservation

An Olympus IX-70 inverted microscope fitted with the Integra micromanipulation station was used for isolation and selection of sperm from the epididymal, testicular or semen sample. A series of 5 µl drops of modified mHTF /HSA were pipetted on an ICSI dish (Falcon 1006 Petri Dish) and overlaid with oil. An aliquot of the sperm specimen was serially diluted in drops until it could be easily screened for the presence of sperm. Motile or twitching sperm were identified and moved to a clean 1 µl drop of mHTF/HSA using an ICSI needle. In patients with severe impairment of spermatogenesis, the screening process could take as long as 4 h Attention was paid to sperm head morphology during the selection process. Sperm heads were cleared of any surrounding debris by repeated aspiration and expulsion from the ICSI needle to allow clear visualization.

For single sperm cryopreservation, 10–20 of the selected sperm were moved to a 1 µl drop containing a 1:1 mixture of medium and TYB-GLY overlaid with oil. The sperm were allowed to equilibrate for 30 min in cryoprotectant solution. The dish was then moved to a dissecting scope. The sperm were visualized using darkfield microscopy, with the light set at maximum illumination. The freezing vessel was the two piece High Security Straw (HSV), consisting of an outer straw and a thin capillary tube with a pre-formed gutter. Using a very finely drawn glass micropipette, sperm were drawn into the tip and then deposited on the capillary tube. Care was taken to load the sperm in less than 1.0 µl of fluid .and place it in the gutter closest to the open end of the capillary tube. The capillary tube was then carefully inserted in to the HSV outer straw and the flared end was sealed off. To allow slow cooling, the HSV straw was placed in a Styrofoam box with a depression cut in it to hold the straw. The box was cooled at −20°C for 1 h and the straw was then plunged in to liquid nitrogen.

Thawing of sperm from the HSV straw was also done with the aid of a dissecting scope. A 1 µl drop of modified HTF-Hepes /HSA was placed in the center of an ICSI dish. The HSV straw was cut and the inner capillary rod with sperm was quickly brought into focus under the dissecting scope. Before the drop could thaw, it was pushed out on to the surface of the ICSI dish using a finely drawn micropipette. Any remaining fluid in the channel of the capillary rod was also aspirated and added to the microdrop on the plate. The dish was then overlayed with oil and sperm were allowed to settle for 10 min. Moving to the inverted microscope, individual sperm were visualized at 300× magnification and moved from the CPA-containing drop to the one microliter drop of mHTF-Hepes medium. Test-thaws were performed in instances where more than one straw was frozen for a patient. The main outcome measures were recovery rate and demonstration of some degree of motility. These data were important in validating this freezing technique for small numbers of sperm.

#### IVF cycle with intracytoplasmic sperm injection

Basic protocols for ovulation induction, oocyte retrieval, embryo evalaution and embryo transfer have been previously published [4]. Mature metaphase II oocytes were injected with thawed sperm from an epididymal aspiration, testicular sperm preparation or semen specimen. Immature oocytes were allowed to mature to overnight before injection. Oocytes were preferentially injected with sperm frozen in conventional vials. If enough distinctly motile /twitching sperm could not be identified, the HSV straw with individually selected sperm was thawed for the ICSI procedure.

## Results

A total of six men with azoospermia had single sperm cryopreservation in addition to freezing aliquots of their testicular or epididymal sperm in traditional cryovials. Two patients with severe oligospermia were also offered single sperm freezing and these cases are included in this initial data set. The extremely compromised nature of the samples made objective quantitation difficult. The initial evaluation was based on the examination of a 5 µl sample. The number of motile sperm per high power field was estimated. In cases of severely impaired spermatogenesis, reporting was based on the number of motile sperm observed in the entire 5 µl drop. If no motile sperm could be identified, additional aliquots were screened to make some approximation of viable sperm in the sample. Table 1 details the type of sperm and their pre-and post-thaw characteristics.

**Table 1:** Single sperm freezing on the HSV straw

			Cryo Vial	Single Sperm Cryopreservation			
Case ID	Sperm type	Initial assessment (motile sperm)	Pre-treatment test-thaw (motile sperm )	# of sperm loaded on HSV	Total sperm recovered	Sperm with motility	Comments
1	TESB	1–4 per 5 µl	Rare twitching*	15	9	0	Fert 3/5, ± ET(1)
2	PESA	300/hpf	40/hpf	22	14	12	Fert 2/5
3	TESB	2–5 per 5 µl	2–3 twitching in 5 µl drop	16	16	16	Fert 0/2
4	PESA	10–15/hpf	2 twitching in 5 µl drop	15	9	7	
5	TESB	2–5 per 5 µl	1 twitching in 10 µl drop	10	10	10	
6	TESB	Rare motile*	Rare twitching*	1	1	1	Fert 1/1, ± ET,(1) Live birth
7	SEMEN	5–8 twitching/hpf	2 twitching per 5 µl drop	15	12	7	Fert 1/2, ± ET(1)
8	SEMEN	37 motile in 2 µl pelleted sample	1 twitching per 5 µl drop	15	5	3	Fert 2/2, ± ET(1) ET

Describes the source and characteristics of the sperm specimens, as well as pre and post-thaw assessment after conventional freezing. Details regarding the number of sperm loaded during Single Sperm Freezing (SSF), recovery and observed motility are also shown

*Fert* fertilization rate after ICSI of mature oocytes; *ET* number of embryos derived from SSF that were transferred;

*Rare motile/twitching identified after screening 35 µl sample.

The methodology for selecting and loading sperm onto the HSV straw was extremely simple and did not require any special equipment, beyond a micromanipulation set up. Use of darkfield illumination facilitated visualization of the sperm during the loading step. In all cases, the ~0.5 µl of fluid containing sperm was quite easily unloaded from the capillary gutter of the HSV straw during thawing. Sequestration of the sperm in this tiny fluid volume, rather than immersion of the capillary tube into medium, during the thawing step was critical for successful recovery of sperm. Immediately overlaying with oil prevented evaporation of fluid. The recovery of individually selected and frozen sperm from the HSV straw ranged from 33% (5 sperm from 15 loaded) to 100% (one sperm loaded and recovered). Specific details for each case are presented in Table 1. Motile or twitching sperm were observed after thawing in all but one case.

Our first test case resulting in a successful pregnancy is also presented in this report. This was a 35 year old male with non-obstructive azoospermia undergoing a testicular biopsy to retrieve sperm for an ICSI cycle. Tissue from the right testis yielded no sperm. Tissue from the left testis was extensively screened by two technicians to locate sperm. Rare motile sperm were observed after examination of multiple aliquots of the initial testicular preparation. During this initial screening, a single sperm with good head morphology and distinct motility was identified and extracted away from the testicular homogenate, containing red blood cells and disrupted testicular cells. This single sperm was cleared free of debris and frozen on an HSV straw. The remaining testicular homogenate was frozen in cryovials after the assessment was completed.

**Fig. 1:**
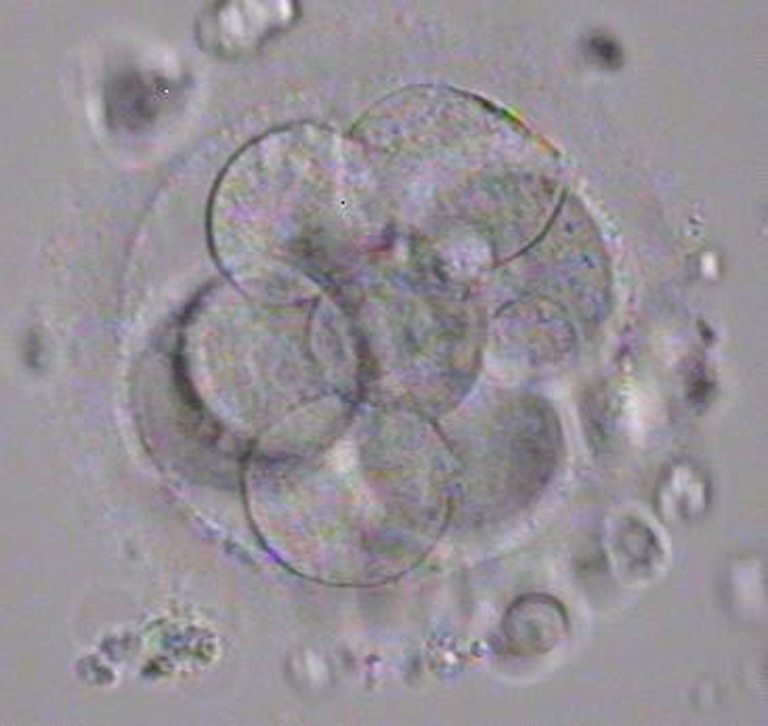
Single embryo transferred on Day 3 at the 8-cell stage, resulting in our first live birth with the Single Sperm Freezing (SSF) technique

The couple were counseled as to the poor quality of the testicular sperm sample but elected to attempt a cycle of IVF/ICSI. The patient was also consented for egg freezing if not enough sperm were found. Eight mature oocytes were retrieved. Vials containing the testicular extract were first thawed. The sample was methodically screened by 2–3 embryologists for over 3 h to isolate sperm for the ICSI procedure (~9 man-hours of searching). The entire thawed testicular digest yielded only one sperm with questionable motility. A single oocyte was injected but the oocyte subsequently degenerated. We went on to thaw the single sperm frozen on the HSV straw. This singly frozen sperm was easily recovered and exhibited slight swaying motion. It was used to inject a second oocyte and resulted in a normal zygote with two pronuclei, the following morning. All remaining oocytes were frozen. The zygote from single sperm freezing went on to form a morphologically normal 8-cell embryo with minimal fragmentation by Day 3 of culture (Fig. 1). The wife, a 32 year old, had a single embryo transfer (SET) and a clinical pregnancy was established. The patient delivered a healthy baby girl who is presently 9 months old.

## Discussion

This is the first description of a technique for freezing individually selected sperm in microquantities of fluid in a High Security Straw (HSV). The device is a closed system for cryopreservation and has FDA clearance. We report on the first pregnancy and delivery with this new and unique methodology for single sperm cryopreservation. This success is truly phenomenal if we give consideration to the fact that the entire testicular preparation ultimately yielded only two sperm. That the lone frozen sperm had the vitality to create a baby , speaks to the potential of this technique.

Cryopreservation of surgically retrieved sperm from the testis and epididymis is an integral part of the effective management and treatment of severe male infertility in ART programs around the country. The low sperm counts, weak motility and contamination of samples with red blood cells and cellular debris make successful cryopreservation and recovery of viable sperm from such samples a challenge. There is a clear need for a simple, easily implemented and safe closed system for freezing individual spermatozoa.

The concept of single sperm freezing, first introduced by Cohen and colleagues in [3], with the freezing of sperm in hamster zonae has not really taken off primarily due to difficulties in finding a suitable vessel for freezing miniscule fluid volumes with 5–20 sperm. The HSV straw allows this to be accomplished in a non-biologic vessel without direct contact with liquid nitrogen. It should also be emphasized that the sperm are being slow frozen and not vitrified. This distinction is important as there is currently little data on sperm vitrification and live birth rates. Our methodology uses conventional test-yolk buffer:glycerol cryoprotectant combined with slow cooling. The limited data set presented in this report and the case study provide the basis for further evaluation of the potential of this technique in the most severe male factor patients. The technique could also potentially benefit patients with severe oligospermia and only rare sperm in their ejaculate. Clearly , more extensive study is needed to validate this technique and to determine its efficacy for single sperm freezing.
